# Emergency medical team coordination management following the 2024 Vanuatu earthquake

**DOI:** 10.5365/wpsar.2026.17.2.1312

**Published:** 2026-06-30

**Authors:** Sharin Vile, Jimmy Obed, Pierre-Yves Beauchemin, Samuel Kemuel, Tatsuhiko Kubo, Erin E Noste, Chandra Gilmore, Philippe Guyant, Ryusuke Ikeda, Tomoaki Natsukawa, Kaoru Harada, Yuki Takamura, Hideshige Tanaka, Hirotaka Sugiyama, Sean T Casey

**Affiliations:** a Ministry of Health, Port Vila, Vanuatu.; b World Health Organization Regional Office for the Western Pacific, Manila, Philippines.; c Department of Public Health and Health Policy, Graduate School of Biomedical and Health Sciences, Hiroshima University, Hiroshima, Japan.; d Department of Multi-Sectoral Preparedness & Strategic Coordination for Health Security, Center for Health Security, Graduate School of Medicine, Kyoto University, Kyoto, Japan.; e Department of Emergency Medicine, University of California San Diego, San Diego, California, United States of America.; f World Health Organization Division of Pacific Technical Support, Suva, Fiji.; g World Health Organization Country Liaison Office, Port Vila, Vanuatu.; h Secretariat of Japan Disaster Relief / Japan International Cooperation Agency, Tokyo, Japan.; i Yodogawa Christian Hospital, Osaka, Japan.; j Seirei Mikatahara General Hospital, Hamamatsu, Shizuoka, Japan.

## Abstract

**Problem:**

On 17 December 2024, a magnitude 7.3 earthquake struck the Pacific Island country of Vanuatu, affecting over 80 000 people and causing 14 deaths and 265 injuries. Facing limited specialist clinical capacity, increased patient load and strained facilities, Vanuatu’s Ministry of Health requested support from emergency medical teams.

**Context:**

Vanuatu, highly disaster-prone and geographically dispersed, has a health system constrained by limited human resources and recent cyclone-related damage. The country’s national emergency medical team, the Vanuatu Medical Assistance Team, was established in 2017 and plays a central role in health emergency response in the country.

**Action:**

Within 24 hours of the earthquake, the Vanuatu Medical Assistance Team was activated and established an Emergency Medical Team Coordination Cell within the national health emergency operations centre. National and international emergency medical teams from Australia, Fiji, Indonesia, Japan and New Zealand were deployed over the subsequent weeks and provided clinical, mental health and information management support. The World Health Organization’s emergency medical team Minimum Data Set was implemented to monitor health trends and inform response actions.

**Outcome:**

Eight national and international emergency medical teams deployed alongside local health services. Together, they reported more than 5500 consultations to the Emergency Medical Team Coordination Cell, and a specialized team provided mental health and psychosocial support to 521 people. Minimum Data Set analysis showed a rapid decline in trauma-related presentations and detected increases in influenza-like illness, guiding the transition from emergency to recovery.

**Discussion:**

This response underscored the value of emergency medical teams following disasters, the importance of national leadership and coordination, and the significance of regional solidarity and partnerships. It also highlighted the importance of information management during emergencies and demonstrated potential innovations in this space.

## PROBLEM

**Fig. 1:**
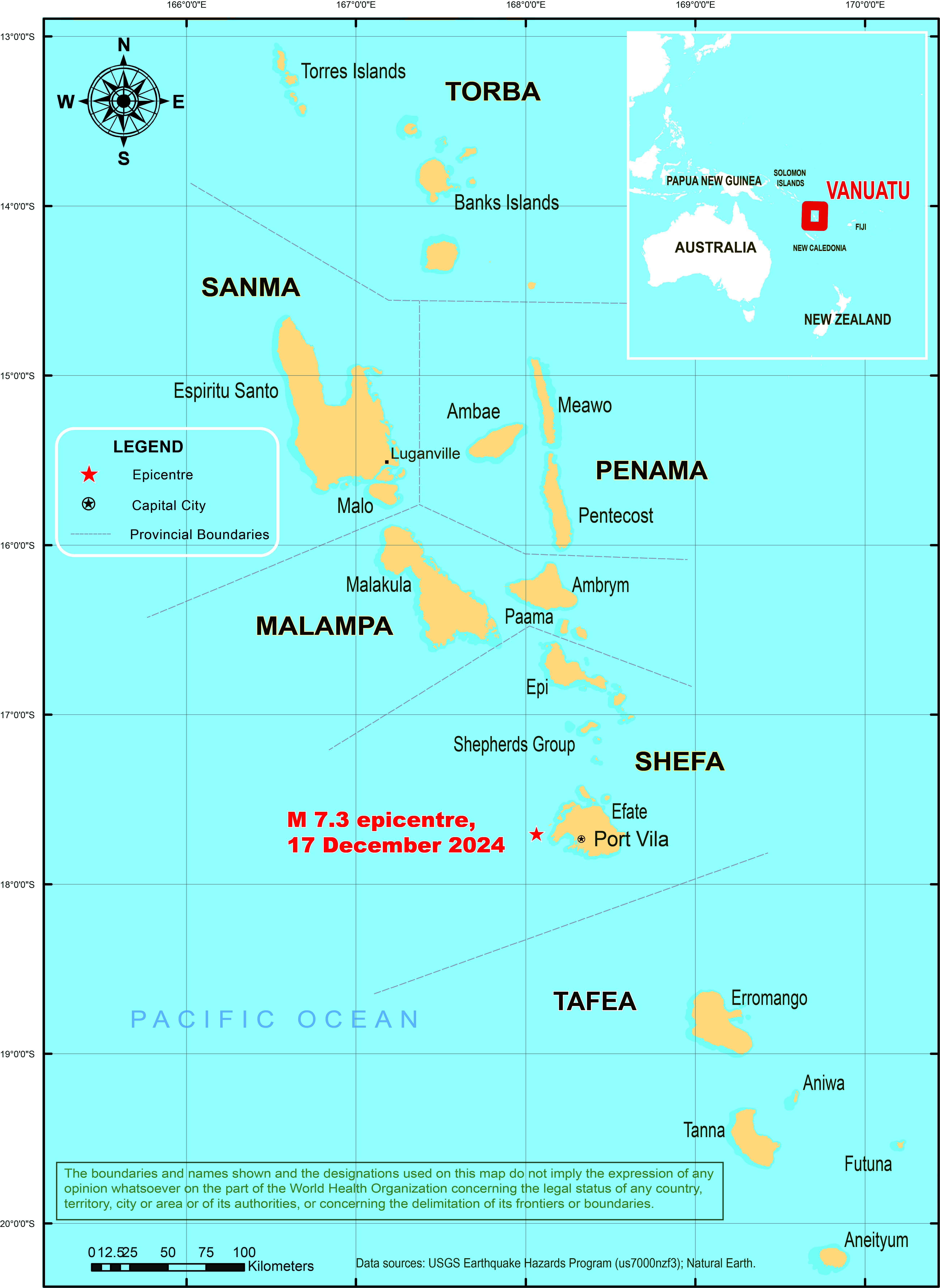
Map of Vanuatu showing the epicentre of the magnitude 7.3 earthquake of 17 December 2024, with provincial boundaries and regional context (inset)

The Pacific Island nation of Vanuatu was struck by a magnitude 7.3 offshore earthquake on 17 December 2024, approximately 30 km west of the capital city of Port Vila (**Fig. 1**). (*1*) This disaster affected over 80 000 people in and around Port Vila, leaving over 10 buildings destroyed and around 570 houses damaged; Vanuatu’s national referral hospital, Vanuatu National Hospital (VNH), was also damaged. (*1*, *2*) The earthquake caused 14 deaths and approximately 265 injuries, and displaced approximately 2435 individuals across six evacuation centres and host families. (*1*, *2*) With limited specialist clinical capacities and an overwhelmed and damaged hospital in the capital city, the Government of Vanuatu, through its Ministry of Health (MoH) and Ministry of Foreign Affairs, requested assistance from international emergency medical teams (EMTs). (*2*)

[**insert** Figure 1]

## CONTEXT

Vanuatu, an archipelago of 83 islands (65 inhabited) in the South Pacific, is among the world’s most disaster-prone nations due to its location along the Pacific Ring of Fire and within the South Pacific cyclone belt. Its population of 300 000 is spread across four archipelagos, covering over 663 000 km^2^ of ocean territory. (*3*) Vanuatu’s geography makes the country highly vulnerable to natural hazards, including earthquakes, tsunamis, tropical cyclones, flooding and volcanic eruptions. (*4*)

Vanuatu is classified by the World Bank as a lower-middle income country, with life expectancy of 74.2 for females and 71.6 for males. (*3*) The country faces a marked shortage of health professionals and capacity, with 0.17 physicians per 1000 people. (*5*) Noncommunicable diseases (NCDs) account for up to 74% of all deaths, while common infectious diseases include leptospirosis, dengue and tuberculosis. (*6*) Vanuatu’s health system includes six hospitals (including two tertiary referral hospitals), 35 health centres, and approximately 290 aid posts and dispensaries. (*7*) It faces significant workforce limitations, including shortages of specialist physicians and loss of health workers to overseas employment and retirement. (*7*) At the time of the earthquake, Vanuatu had no orthopaedic surgeons, one psychiatrist and a significant shortage of nurses. The nursing school in Vanuatu has not been enrolling or graduating nurses since 2022 due to an internal audit conducted by the Vanuatu Qualification Authority to evaluate compliance and national standards. Beyond its chronic challenges, Vanuatu’s health system was already recovering from multiple disasters in 2023, including back-to-back tropical cyclones. (*8*) A 2025 Joint External Evaluation of Vanuatu’s International Health Regulations (2005) core capacities confirmed significant gaps in health emergency response readiness, with both the ability to surge health workforce during public health events and health emergency logistics scoring at the lowest level (1 out of 5). (*9*)

Vanuatu’s MoH established the Vanuatu Medical Assistance Team (VanMAT) in 2017 due to the persistent threat of disasters and outbreaks in the country. (*8*, *10*) The establishment of VanMAT was accomplished with technical and operational support from the World Health Organization (WHO), and with funding and technical support from the Governments of Australia and New Zealand. (*10*, *11*) VanMAT was established within Vanuatu’s MoH, with coordination linkages to Vanuatu’s National Disaster Management Office (NDMO). In peacetime, VanMAT maintains readiness through training and coordination with MoH, NDMO, WHO and regional partners. VanMAT’s EMT Coordination Cell (EMTCC) serves as the national coordination mechanism for EMT deployment during health emergencies, responsible for receiving and processing offers of international assistance, tasking (assigning) EMTs to work in specific affected areas, and consolidating health data to guide response decisions.

VanMAT is one of many national and international EMTs in WHO’s Western Pacific Region, established based on the principles and standards detailed in WHO’s *Classification and minimum standards for emergency medical teams*. (*10*, *12*) EMTs provide clinical care and public health assistance to populations affected by disasters, outbreaks and other health emergencies. VanMAT, like other national EMTs in the South Pacific, was designed by adopting and adapting global EMT standards to Vanuatu’s unique island context and high vulnerability to natural hazards. (*8*) EMTs have been established across the Western Pacific Region, with 16 teams classified (quality-assured) by WHO and peer EMTs by the end of 2024 (of 53 globally). (*10*)

## ACTION

Following the 17 December 2024 earthquake, Vanuatu’s MoH and partners immediately undertook damage and needs assessments at VNH and other health centres. (*1*, *2*) Power and telecommunications were disrupted across Port Vila, with early coordination relying on Starlink satellite connectivity, in-person EMTCC situation updates and bilateral partner channels. Given the uncertainty over the structural integrity of health facilities, a lack of specialist capacity in Vanuatu, and the large number of injured and displaced people, Vanuatu’s MoH requested support from international EMTs in the Western Pacific Region.

On 18 December, VanMAT leadership within the MoH established an EMTCC. On the same day, the Australian Medical Assistance Team (AUSMAT) and New Zealand Medical Assistance Team (NZMAT) deployed forward teams as part of larger support provided by the Governments of Australia and New Zealand. (*13*) The NZMAT forward team demobilized a few days later, while AUSMAT deployed a second rotation of 13 team members, focusing support on VNH.

Additional teams, including VanMAT’s clinical team, were activated in the 10 days after the earthquake to provide specialized support to the response. On 21 December, following an online meeting between Vanuatu’s MoH and the Secretariat of the Japan Disaster Relief Team (JDR)/Japan International Cooperation Agency (JICA), JICA initiated remote support for information management (IM) for Vanuatu’s EMTCC, with team members providing in-person IM support from 26 December, applying WHO’s EMT Minimum Data Set (MDS). (*14*) On 27 December, the Pasifika Medical Association Medical Assistance Team (PACMAT) arrived in Port Vila, deploying as a specialized care team with a focus on mental health and psychosocial support (MHPSS). (*15*) The Indonesia EMT arrived on the same day to establish an outpatient field clinic at Vanuatu’s NCD Hub. The Fiji Emergency Medical Assistance Team (FEMAT) deployed a 12-member team from 5 to 17 January 2025. FEMAT’s response focused on supporting Vanuatu’s NCD Hub, providing outpatient care and supporting haemodialysis at VNH along with AUSMAT.

While some EMTs deployed quickly, others arrived approximately 2 weeks after the earthquake, by which time acute cases related to the earthquake had already been managed. However, Vanuatu’s fragile and understaffed health system required surge support throughout the response and recovery periods.

## OUTCOME

**Fig. 2:**
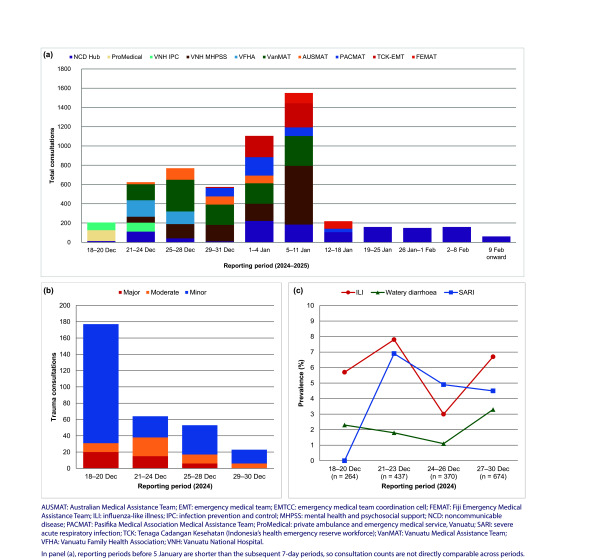
EMT Minimum Data Set summary, Vanuatu earthquake response, December 2024-February 2025: (a) weekly consultations reported to the EMTCC by EMTs and participating local health facilities, by reporting entity; (b) trauma consultations by severity; and (c) infectious disease prevalence among reported cases

VanMAT was activated immediately after the earthquake and led the clinical response, with the overarching health response coordinated by the MoH, supporting the deployment of eight national and international EMTs. From December 2024 to February 2025, more than 5500 consultations were reported to the EMTCC through the MDS by EMTs and participating local health facilities (**Fig. 2**). An MHPSS EMT was also deployed and provided care for 521 individuals, all of whose presentations were directly linked to the earthquake. (*15*) Data collection and information management were supported by JICA, applying the WHO MDS. (*14*)

[**insert** Figure 2]

EMTs provided a wide range of services, including clinical support at VNH, NCD Hub and community locations around Port Vila, as well as rehabilitation, orthopaedic care and MHPSS. In particular, teams contributed specialists who were not readily available in-country, such as orthopaedic surgeons, rehabilitation professionals and mental health specialists. (*13*)

The MoH initiated daily reporting using the EMT MDS just 1 day after the disaster. A version of the MDS was also developed specifically for MHPSS services, adapted from the Japanese national EMT daily MHPSS reporting template. These tools enabled monitoring of disease trends and supported transition planning. Collaborative analysis of MDS data by the EMTCC and JICA IM team showed that trauma-related consultations declined sharply over the first weeks of the response, indicating a transition from acute emergency to recovery. MDS syndromic surveillance, cross-referenced with the national early warning, alert and response system, detected increases in influenza-like illness presentations following the earthquake, enabling early public health action. The MDS was also extended beyond EMTs for use by local health facilities, a novel approach that broadened the scope of routine health data collection during the response.

Despite strong coordination and data reporting mechanisms, challenges emerged in the operational aspects of the response. Based on observations from the VanMAT coordinator and WHO logistics expert, pre-positioned emergency supplies were not properly stored or inventoried, limiting the ability to rapidly deploy VanMAT’s logistics cache when needed, and VanMAT satellite communication systems were not activated during early blackout periods. Most EMTs deployed outside of their formal WHO classification typologies. For example, AUSMAT (a Type 2 EMT) deployed as a Specialized Care Team to VNH, and PACMAT (pursuing Type 1 Mobile classification) deployed to provide both outpatient care and specialized MHPSS services. (*13*, *15*)

While the Vanuatu MoH coordinated most EMT engagement through the EMTCC, some offers of assistance, both accepted and declined, were managed bilaterally or through diplomatic channels, occasionally outside of EMTCC processes. While the MoH engaged EMTs directly to request their support, offers of assistance were also received by at least two other teams, which were respectfully declined based on an MoH determination that needs were sufficiently addressed, and applying a preference for local teams with whom Vanuatu had existing relationships and for those pursuing WHO EMT classification (**Table 1**).

**Table 1:** EMTs and health emergency surge personnel^a^ deployed in response to the Vanuatu earthquake, 2024–2025

EMT	Deployment period	Deployment location	Team composition (function)	Team composition (gender)
**VanMAT^b^**	**22 December 2024–9 January 2025**	**Vanuatu National Hospital**	**4 registered nurses** **1 midwife** **1 driver**	**4 females, 2 males**
**AUSMAT ** **(Alpha rotation)**	**18 December 2024–6 January 2025**	**Vanuatu National Hospital**	**2 nurses** **1 midwife** **2 medical officers** **1 logistician**	**2 females, 4 males**
**AUSMAT ** **(Bravo rotation)**	**23 December 2024–6 January 2025**	**Vanuatu National Hospital**	**3 logisticians** **1 clinical team lead** **1 general medicine specialist** **1 physiotherapist** **1 wound care nurse** **2 scrub/scout nurses** **1 biomedical engineer** **1 recovery room & general nurse** **1 occupational therapist** **1 general surgeon**	**6 females, 7 males**
**NZMAT**	**18–22 December 2024**	**Ministry of Health/Vanuatu National Hospital**	**1 mission lead** **1 doctor** **1 logistician**	**3 males**
**JDR/JICA ** **(information management support)**	**21 December 2024–18 January 2025**	**EMTCC (in-country and remote support)**	**1 operations manager** **1 team leader** **1 coordinator** **1 deputy coordinator** **5 information management**	**2 females, 7 males** **(3 remote, 6 onsite)**
**UNFPA (midwives)**	**25 December 2024–22 March 2025**	**Vanuatu National Hospital and Vanuatu Family Health Association Mobile team**	**4 midwives**	**4 females**
**PACMAT (MHPSS specialized care team)**	**27 December 2024–17 January 2025**	**Specialized care team**	**1 team lead** **1 logistician** **2 psychiatrists** **2 mental health nurses** **2 registered nurses** **1 medical officer**	**2 females, 7 males**
**Indonesia EMT**	**27 December 2024–10 January 2025**	**Type 1 Mobile (NCD Hub and outreach with Shefa health team)**	**5 nurses** **3 general practitioners** **1 pharmacist** **1 logistics and administration** **1 emergency specialist** **1 internist specialist** **1 orthopaedic** **1 anaesthesia specialist** **1 surgical specialist**	**15 males**
**FEMAT**	**5–17 January 2025**	**Type 1 Mobile (NCD Hub)**	**1 team lead** **1 clinical lead** **1 logistics lead** **1 nursing lead** **3 nurses (paediatric, emergency and haemodialysis)** **1 midwife** **1 carpenter** **1 electrician** **1 biomedical technician** **1 data officer**	**7 females, 5 males**

AUSMAT: Australian Medical Assistance Team; EMT: emergency medical team; EMTCC: emergency medical team coordination cell; FEMAT: Fiji Emergency Medical Assistance Team; JDR: Japan Disaster Relief; JICA: Japan International Cooperation Agency; MHPSS: mental health and psychosocial support; NCD: noncommunicable disease; NZMAT: New Zealand Medical Assistance Team; PACMAT: Pasifika Medical Association Medical Assistance Team; UNFPA: United Nations Population Fund; VanMAT: Vanuatu Medical Assistance Team.

^a^ A nongovernmental organization, Respond Global, also provided support to Shefa Province, but was not considered an EMT in this response.

^b^ VanMAT, Vanuatu’s national EMT, was formally deployed through the EMTCC. VanMAT leadership in Port Vila established the EMTCC on 18 December 2024; the VanMAT clinical team arrived in Port Vila on 22 December 2024.

## Discussion

The Vanuatu earthquake response demonstrated that nationally led coordination, underpinned by structured data collection through the EMT MDS, is central to mobilizing surge clinical capacity and managing the transition from emergency to recovery. Investment in national coordination mechanisms and systematic IM enabled a small island developing state to direct international assistance while maintaining national ownership.

VanMAT’s national EMT activation demonstrated the importance of this national health emergency response capability. Vanuatu scaled up the EMTCC immediately following the earthquake, enabling national authorities to issue bilateral requests for assistance, receive classified EMTs, and gather and analyse data and information from the day after the earthquake through the departure of the last international EMT. Through the EMTCC, the MoH consolidated requests for surge health workforce and tasked EMTs based on needs and available resources, drawing on the MDS to match specialized skills to locations.

EMTs responded quickly to the Government’s call for assistance, mostly through bilateral arrangements. Evidence suggested that EMTs effectively supported response actions, particularly through deployment of clinicians with specialties that are typically not available in Vanuatu. This response highlights the utility of a connected regional EMT network, with broader implications for global preparedness through sharing operational data and lessons across borders. (*10*)

The proportion of clinical consultations directly related to the earthquake quickly decreased, according to MDS data submitted to the EMTCC, guiding the transition from emergency to recovery. The MDS was used for reporting by both EMTs and local health facilities, suggesting the potential for broader application in future health emergency responses. This expansion of the EMT MDS within the existing local health system following the disaster could suggest an opportunity for future use in similar contexts.

Determining the number and types of EMTs needed is often a challenging task because information is often imperfect. The complexity of managing offers of international support, particularly when channelled through bilateral mechanisms, reinforces the importance of clear national coordination structures and broad awareness of EMT coordination processes. However, in Vanuatu, regional solidarity and pre-existing relationships were evident throughout the response and directly facilitated coordination. For example, PACMAT had previously worked with Vanuatu’s mental health services, AUSMAT had deployed to Vanuatu in previous emergencies, and EMTCC leadership had participated in AUSMAT-led training. These relationships enabled rapid tasking and reduced the burden of orientation during international deployments. However, the deployment of EMTs outside of their WHO classified typology, while practical, raises questions about how classification frameworks reflect real-world deployment needs. This warrants further discussion within the global EMT community, the WHO EMT Secretariat and the EMT Strategic Advisory Group. Many EMTs expend significant resources to achieve WHO classification, yet the roles they fulfil may differ from their classified typology. Acknowledging this need for flexibility, while maintaining core EMT principles and minimum standards, may better serve affected populations.

Integrating information and data management from the early stages of a health emergency response can improve data quality and help decision-makers understand the situation more clearly. (*14*) In this event, early remote collaboration with the JDR Secretariat/JICA followed by in-person technical support provided more consistent EMT and response data. An important aspect of IM during this response was collating and interpreting local health facility data alongside EMT reporting. While EMTs contribute to health emergency response, local health providers are the primary responders. Documenting EMT action provides insights into certain elements of the response but may not reflect broader health impacts, including reductions in morbidity, mortality and pressures on resource-limited health systems. Information generated through the EMTCC was shared through WHO EMT coordination mechanisms, reinforcing the value of standardized reporting for cross-border learning.

This study has several limitations. As a descriptive account of a single emergency response, findings may not be generalizable. The analysis relies on aggregated MDS data, which do not capture the full clinical detail of individual patient encounters. The study does not include outcome measures such as mortality or morbidity reduction attributable to EMT interventions. Future research should strive to link EMT operational data with health outcome indicators to strengthen the evidence base.

This response offers lessons for Pacific Island countries and areas on national leadership, coordination, data-driven decision-making and partnerships. It also contributes to the broader global EMT knowledge base through three innovations in data and information management: the extension of MDS reporting to local health facilities beyond EMTs, the adaptation of a Japanese MHPSS reporting template for Pacific use, and the initiation of remote IM support before in-country deployment, as well as the practical deployment of national, classified and non-classified teams in a sudden-onset emergency setting.
